# Determinants of fruit and vegetable intake among 11-year-old schoolchildren in a country of traditionally low fruit and vegetable consumption

**DOI:** 10.1186/1479-5868-3-41

**Published:** 2006-11-24

**Authors:** Asa G Kristjansdottir, Inga Thorsdottir, Ilse De Bourdeaudhuij, Pernille Due, Marianne Wind, Knut-Inge Klepp

**Affiliations:** 1 Unit for Nutrition Research, Landspitali-University Hospital & Department of Food Science, University of Iceland, Reykjavik, Iceland; 2 Department of Movement and Sport Sciences, Ghent University, Ghent, Belgium; 3 Department of Social Medicine, University of Copenhagen, Copenhagen Denmark; 4 Department of Public Health, Erasmus University Medical Centre, Rotterdam, The Netherlands; 5 Department of Nutrition, University of Oslo, Oslo, Norway

## Abstract

**Background:**

Fruit and vegetable consumption is traditionally low in Iceland. The results of the Pro Children cross-Europe survey showed that the consumption was lowest among children in Iceland. The aim of this study was to identify determinants of fruit and vegetable intake among 11-year-old schoolchildren in Iceland.

**Methods:**

A cross-sectional survey was performed in Iceland in the autumn of 2003 as a part of the Pro Children cross-Europe survey. The survey was designed to provide information on actual consumption levels of vegetables and fruits by 11-year-old school children and to assess potential determinants of consumption patterns. A total of 1235 Icelandic children (89%) from 32 randomly chosen schools participated. Hierarchical regression analyses were performed to determine the explained variance of the children's fruit and vegetable intake. In these analyses socio-demographic background variables were entered as a first block, perceived physical-environmental variables as a second block, perceived socio-environmental variables as a third block and personal variables as a fourth block.

**Results:**

64% of the children ate fruit less than once a day, and 61% ate vegetables less than once a day. Respectively, 31% and 39% of the variance in children's fruit and vegetable intake was explained by the determinants studied. About 7% and 13% of the variance in fruit and vegetable intake was explained by the perceived physical-environmental determinants, mainly by availability at home. About 18% and 16% of the variance in fruit and vegetable intake was explained by the personal determinants. For both fruit and vegetable intake, the significant personal determinants were preferences, liking, knowledge of recommendations and self-efficacy.

**Conclusion:**

Interventions to increase fruit and vegetable intake among children should aim at both environmental factors such as greater availability of fruit and vegetables, and personal factors as self-efficacy and knowledge levels concerning nutrition.

## Background

Epidemiological data strongly support a protective effect of regular fruit and vegetable consumption against heart diseases and certain types of cancer [e.g., [1-3]]. The health benefits of fruits and vegetables seen in epidemiology studies are the main reasons for the recommended intake of at least 400 g of fruit and vegetables per day [4]. The Icelandic recommendation is 500 g (> 200 g fruit, > 200 g vegetable), potatoes are excluded, but fruit juice is included in the total. Large population groups, particularly in Northern Europe, however, eat far less than the recommended amount of vegetables and fruits [5]. The consumption is especially low in Iceland among both adults [6,7] and children [8]. It is therefore of public health concern to increase the consumption of fruits and vegetables in the Icelandic population. Increasing fruit and vegetable consumption among children has good potential for improving public health, as healthy food habits acquired in childhood tend to continue into adulthood [9,10]. Moreover, health-related habits are not as firmly rooted in children as adults, which makes them more flexible to change [11]. The school setting provides a valuable opportunity for interventions as a large majority of children can effectively be reached through schools. To develop effective interventions to increase fruit and vegetable consumption, it is essential to know which factors determine the consumption in specific target groups.

Most of the published studies on the determinants of fruit and vegetable intake among children and adolescent have been undertaken in the US, and relatively few studies have been undertaken in Europe [12]. The present study is part of the Pro Children cross-Europe survey, where Iceland is one of 9 participating European countries. The survey was designed to provide information on actual consumption levels of fruits and vegetables in European school children and their parents and to understand the determinants of consumption patterns among children [12]. As fruit and vegetable consumption was lowest among children from Iceland [8], it is of special interest to study the determinants of fruit and vegetable consumption among Icelandic children

In the Pro Children project, constructs from different behavioural theories were included to ensure the inclusion of a large range of potential determinants at the individual, social and environmental level [13]. After reviewing the literature on determinants of fruit and vegetable intake among children and adolescents [14], a conceptual framework was developed in the Pro Children project. The framework proposes that determinants of fruit and vegetable intake can be found in the cultural, physical and social environment, and that they in turn influence the more proximal factors to be found at the personal level [12].

The objective of the present study was to identify determinants of fruit and vegetable intake among 11-year-old schoolchildren in Iceland, the country with the lowest consumption of fruits and vegetables in Europe.

## Methods

### Sample and procedure

The cross-sectional survey, which was conducted October-November 2003, included children in sixth grade (born 1992) and their parents. A random national sample of 33 primary schools was selected from a list from Statistics Iceland. Research clearance was obtained from the Icelandic Data Protection Authority. Approval to contact the schools was obtained from the Reykjavik School District Head Office and Service Centre.

The headmasters of the schools were approached about their willingness to participate, and the class teachers were asked to collect the data using standardized instructions. 32 schools participated, with 1392 sixth graders. The children answered a self-administered questionnaire during one school lesson, with instructions and help from the teacher. A total of 1235 children attending this specific school lesson, turned in the questionnaire (response rate 89%). All students and their parents received written information on the project. Completion of the questionnaire was voluntary and parents could demand that their child's questionnaire not be used in the study. A total of 17 questionnaires were excluded from the analysis due to incomplete answers in the dietary part of the questionnaire, i.e., information on the usual intake of both fruits and vegetables was missing. A further 15 questionnaires were excluded at a parent's request, along with five outliers (fruit and vegetable intake higher than 1500 g per day according to the 24-hour recall) and 19 because information on gender was missing. The final sample consisted of 1179 children, 560 girls and 619 boys. The average age was 11.3 years.

The children were asked about their parents' occupation, and these answers were used as an approximate measure of socioeconomic status (SES). These answers were coded into 5 categories according to a standardized protocol with national adaptation, using the occupation-status list from Statistic Iceland (ISTARF 95).

### Instrument

A self-administered questionnaire was developed to assess fruit and vegetable intake among children and to identify the determinants of their fruit and vegetable intake. The dietary part of the questionnaire was composed of a precoded 24-hour recall part and a food frequency part (both parts self-administered). The precoded 24-hour recall part of the Questionnaire was included to give information about intake of the group and the amount and types of fruits and vegetables, whereas the food frequency part made it possible to rank individuals according to levels of usual intake. The food frequency questionnaire (FFQ) included four questions on usual intake (over the last month) of; fresh fruits, salad or grated vegetables, other raw vegetables and cooked vegetables (not including potatoes). Fruit juice was not included. The response categories ranged from never to every day and more than twice a day. The dietary part of the questionnaire was found to be reliable and valid [15,16].

The determinant part of the questionnaire aimed at measuring 14 constructs that were analogous for fruit and for vegetable intake: knowledge of recommendations, attitudes, liking, general self-efficacy, intention, habit, preferences, perceived barriers, modelling, active parental encouragement, family rules related to demands and allowances, availability at home and availability at school. Examples of questions: "Are there usually different kind of fruits available at home?", "Is there usually fruit available at home that you like?", "Do your parents demand that you eat fruit every day?" and "Are you allowed to eat as much fruit as you like?" the response categories for these questions, ranging from yes always to never [further see appendix in [17]]. The determinant part of the questionnaire was found to be reliable and valid [13].

### Statistical analysis

The food frequnecy questionnaire was used for analysis in this paper, not the 24-hour recall. The vegetable intake scale from the food frequency questionnaire consisted of three questions, that were recoded to take different portion sizes into account before they were summed. Standardized portion sizes were used, that had been validated before [15,16].

The socio-demographic background variables used were gender, residence (living in Reykjavik or in other regions of the country) and socioeconomic status. A total of 146 questionnaires had missing or insufficient information to code the socioeconomic status of the mother, 191 to code the SES for father, and 291 for either mother or father. While this missing value decreased the number included in the regression analysis, it did not alter the findings presented in this paper (analyses not shown).

Regression assumptions regarding normality, linearity and homoscedasticity were found to be acceptable, and parametric statistics were therefore used. Non-parametric tests were also applied, but the results did not differ from the parametric tests, and are therefore not reported. Pearson correlation coefficients were calculated to show the non-adjusted relationship between each variable. Hierarchical regression analyses were performed to determine the explained variance of the children's fruit and vegetable intake. In the hierarchical regression analyses, socio-demographic background variables were entered as a first block, perceived physical-environmental scales as a second block, perceived socio-environmental scales as a third block and personal scales as a fourth block. Standardized regression coefficients (Beta) are given for each scale. For each step, the adjusted variance explained (adj. R^2^) and the change of variance explained in each step (R^2 ^change) are given.

Analyses were conducted using SPSS version 11 (SPSS inc. 1999). A p-value ≤ 0.05 was considered to be significant.

## Results

Mean values for all independent variables by gender are presented in Table 1. The distribution of the intake was skewed as a large part of the group was low in consumption levels. 64% of the children ate fruit less than once a day, and 61% ate vegetables less than once a day. The mean usual frequency of both fruit and vegetable intake was higher among girls than boys. There were generally higher scores on the fruit scales than on the vegetable scales, i.e. children had more positive attitude towards fruit than vegetables. Girls had significantly higher scores on the following fruit determinant scales: modelling, demanding family rule, knowledge of recommendations, attitudes, liking, self-efficacy, preferences, perceived barriers (all t > 2.0, p < 0.05). Girls had significantly higher scores on the following vegetable determinant scales; availability at home, modelling, demanding family rule, allow family rule, attitudes, liking, self-efficacy, perceived barriers (all t > 2.4, p < 0.05). There was non significant difference between genders on the other scales. Cronbach's alpha were acceptable (alpha ranging from 0.59 to 0.87) for all scales except self-efficacy (fruit intake alpha 0.44). All independent scales were significantly correlated to children's fruit and vegetable intake, with the exception of the relationship between availability at school and fruit intake (Tables 2 and 3).

**Table 1:** Mean values, standard deviation (SD) for girls and boys separately and combined and Cronbach's alpha values.

			**Fruit**				**Vegetables**			
**Scale**	**Items**	**Range**	**Girls Mean (SD)**	**Boys Mean (SD)**	**All Mean (SD)**	Cronbach's alpha	**Girls Mean (SD)**	**Boys Mean (SD)**	**All Mean (SD)**	Cronbach's alpha
Intake (FFQ)		8-point scale converted into times per day	1.00 (0.81)	0.84 (0.78)	0.91 (0.80)	-	1.17 (1.16)	0.85 (0.95)	1.00 (1.06)	-
*Personal*										
Knowledge of recommendations	1	8-point scale recoded (0) less than twice a day (1) twice a day or more	0.56 (0.50)	0.50 (0.50)	0.53 (0.50)	-	0.39 (0.49)	0.36 (0.48)	0.38 (0.48)	-
Attitudes	2	5-point scale from (2) I fully agree to (-2) I fully disagree	1.25 (0.73)	1.05 (0.90)	1.15 (0.83)	0.69	0.94 (0.93)	0.79 (1.03)	0.86 (0.99)	0.80
Liking	2	5-point scale from (2) I fully agree to (-2) I fully disagree	1.59 (0.54)	1.34 (0.82)	1.46 (0.71)	0.71	1.06 (0.98)	0.85 (1.08)	0.95 (1.04)	0.83
Self-efficacy	2	5-point scale from (2) I fully agree to (-2) I fully disagree	1.30 (0.80)	1.09 (0.93)	1.19 (0.88)	0.44	1.12 (0.90)	0.85 (1.00)	0.97 (0.96)	0.80
Preferences	12	5 point scale from (2) like very much to (-2) dislike very much	1.27 (0.48)	1.13 (0.61)	1.19 (0.56)	-	0.48 (0.71)	0.42 (0.80)	0.45 (0.75)	-
Perceived barriers	4	5-point scale from (2) I fully agree to (-2) I fully disagree	-1.64 (0.55)	-1.37 (0.78)	-1.50 (0.69)	0.73	-1.52 (0.68)	-1.23 (0.91)	-1.37 (0.82)	0.80
*Perceived socio-environmental*										
Modelling	3	5-point scale from (2) I fully agree to (-2) I fully disagree	0.61 (0.71)	0.49 (0.71)	0.54 (0.71)	0.59	0.52 (0.72)	0.39 (0.73)	0.45 (0.73)	0.68
Active parental encouragement	2	5-point scale from (2) I fully agree to (-2) I fully disagree	0.65 (1.09)	0.53 (1.19)	0.58 (1.14)	0.80	0.36 (1.08)	0.26 (1.23)	0.30 (1.16)	0.87
Demand family rule	1	5 point scale (2) yes always to (-2) never	0.32 (1.14)	0.04 (1.24)	0.18 (1.20)	-	0.08 (1.13)	-0.09 (1.24)	-0.01 (1.19)	-
Allow family rule	1	5 point scale (2) yes always to (-2) never	1.71 (0.70)	1.62 (0.77)	1.66 (0.74)	-	1.52 (0.78)	1.30 (1.01)	1.40 (0.91)	-
*Perceived physical-environmental*										
Availability at home	3	5 point scale (2) yes always to (-2) never	1.01 (0.62)	0.94 (0.68)	0.97 (0.65)	0.59	0.82 (0.72)	0.70 (0.84)	0.76 (0.78)	0.68
Availability at school	1	5 point scale (2) yes always to (-2) never	-0.64 (1.52)	-0.75 (1.50)	-0.69 (1.51)	-	-1.02 (1.32)	-1.11 (1.22)	-1.06 (1.27)	-

**Table 2:** Pearson correlation between all variables, fruit intake and it's determinants.

	**1**	**2**	**3**	**4**	**5**	**6**	**7**	**8**	**9**	**10**	**11**	**12**	**13**
**1 **Fruit intake (FFQ)	1												
*personal*													
**2 **Knowledge	0.29**	1											
**3 **Attitudes	0.33**	0.20**	1										
**4 **Liking	0.39**	0.19**	0.60**	1									
**5 **Self-efficacy	0.39**	0.14**	0.37**	0.49**	1								
**6 **Preferences	0.33**	0.07**	0.33**	0.49**	0.34**	1							
**7 **Perceived barriers	-0.26**	-0.13**	-0.31**	-0.45**	-0.38**	-0.24**	1						
*perceived socio-environmental*													
**8 **Modelling	0.23**	0.13**	0.27**	0.28**	0.18**	0.16**	-0.10**	1					
**9 **Active encouragement	0.17**	0.10**	0.31**	0.31**	0.16**	0.18**	-0.16**	0.38**	1				
**10 **Demand family rule	0.27**	0.16**	0.27**	0.29**	0.22**	0.16**	-0.15**	0.36**	0.65**	1			
**11 **Allow family rule	0.11**	0.06	0.11**	0.14**	0.17**	0.06*	-0.13**	0.11**	0.20**	0.23**	1		
*perceived physical-environmental*													
**12 **Availability at home	0.28**	0.11**	0.25**	0.30**	0.32**	0.20**	-0.25**	0.28**	0.31**	0.36**	0.31**	1	
**13 **Availability at school	0.02	0.03	0.04	0.05	0.02	0.05	0.02	0.09**	0.06*	0.04	0.04	0.08**	1

FFQ- food frequency questions

* Correlation significant at the 0.05 level (two tailed)

**Correlation significant at the 0.01 level (two tailed).

**Table 3:** Pearson correlation between all variables, vegetable intake and it's determinants.

	**1**	**2**	**3**	**4**	**5**	**6**	**7**	**8**	**9**	**10**	**11**	**12**	**13**
**1 **Vegetable intake (FFQ)	1												
*Personal*													
**2 **Knowledge	0.22**	1											
**3 **Attitudes	0.40**	0.27**	1										
**4 **Liking	0.46**	0.22**	0.72**	1									
**5 **Self-efficacy	0.40**	0.18**	0.46**	0.53**	1								
**6 **Preferences	0.45**	0.14**	0.44**	0.55**	0.40**	1							
**7 **Perceived barriers	-0.33**	-0.13**	-0.40**	-0.46**	-0.46**	-0.31**	1						
*perceived socio-environmental*													
**8 **Modelling	0.28**	0.09**	0.30**	0.28**	0.24**	0.22**	-0.16**	1					
**9 **Active encouragement	0.28**	0.09**	0.40**	0.35**	0.24**	0.26**	-0.16**	0.51**	1				
**10 **Demand family rule	0.35**	0.10**	0.33**	0.29**	0.23**	0.24**	-0.16**	0.41**	0.70**	1			
**11 **Allow family rule	0.19**	0.09**	0.23**	0.23**	0.23**	0.15**	-0.13**	0.20**	0.24**	0.26**	1		
*perceived physical-environmental*													
**12 **Availability at home	0.38**	0.10**	0.36**	0.39**	0.40**	0.30**	-0.30**	0.36**	0.37**	0.45**	0.43**	1	
**13 **Availability at school	0.07	-0.02	0.07*	0.07**	0.07*	0.08**	-0.04	0.11**	0.07**	0.07*	0.03	0.08**	1

FFQ- food frequency questions

* Correlation significant at the 0.05 level (two tailed)

**Correlation significant at the 0.01 level (two tailed).

The regression model explained 31% of the variance in children's fruit intake (Table 4). A similar pattern was seen for boys and girls separately, but social and personal variables explained more of the variance among girls; the model explained 39% of the variance among girls, and less for boys (24%). About 2% of the variance was explained by background variables, gender contributed significantly, but gender became non-significant in the last step when personal scales were added. Modelling was significant for girls separately (p = 0.02), also when personal scales were added to the model, but non-significant for boys (p = 0.39). About 18% of the variance was explained by the personal scales, self-efficacy, knowledge of recommendations, liking and preferences contributed significantly. All of these scales were positively related to fruit intake. Attitudes, and perceived barriers did not contribute significantly to the explanation; for girls separately perceived barriers were negatively related to fruit intake (p = 0.02), but non-significant for boys (p = 0.82).

**Table 4:** Hierarchical regression model explaining the variance in children's fruit intake (*n *= 765).

	step 1		step 2		step 3		step 4	
	Beta	P-value	Beta	P-value	Beta	P-value	Beta	P-value
*background variables*								
Gender	0.12	< 0.01	0.10	< 0.01	0.08	0.02	0.03	0.38
Residence	0.03	0.46	0.05	0.15	0.05	0.12	0.03	0.32
SES mother	0.07	0.08	0.06	0.11	0.06	0.11	0.01	0.71
SES father	0.06	0.09	0.04	0.25	0.04	0.23	0.04	0.22
*perceived physical-environmental*								
Availability at home			0.27	< 0.01	0.20	< 0.01	0.08	0.04
Availability at school			0.00	0.90	-0.01	0.74	-0.01	0.77
*perceived socio-environmental*								
Modelling					0.14	< 0.01	0.07	> 0.05
Active encouragement					-0.11	0.01	-0.14	< 0.01
Demand family rule					0.21	< 0.01	0.16	< 0.01
Allow family rule					-0.01	0.83	-0.04	0.25
*personal*								
Knowledge							0.17	< 0.01
Attitudes							0.04	0.27
Liking							0.12	0.01
Self-efficacy							0.23	< 0.01
Preferences							0.12	< 0.01
Perceived barriers							-0.05	0.17
								
adjusted R^2^	0.019		0.088		0.130		0.312	
R^2 ^change	0.024	< 0.01	0.071	< 0.01	0.046	< 0.01	0.185	< 0.01

The regression model explained 39% of the variance in children's vegetable intake, similarly to fruit intake but environmental variables explained more of the variance in vegetable intake (Table 5). A similar pattern was seen for boys and girls separately, but background and environmental variables explained more of the variance among boys. The model explained 35% of the variance among girls and 38% for boys. About 6% of the variance was explained by the background variables, the father's socioeconomic status and gender. Both the father's and mother's socioeconomic status were positively related to vegetable intake for boys separately (p < 0.01 and p = 0.04), but non-significant for girls (p = 0.12 and p = 0.43). As with fruits, modelling was significant for girls separately (p < 0.01) but not for boys (p = 0.25).

**Table 5:** Hierarchical regression model explaining the variance in children's vegetable intake (*n *= 776).

	step 1		step 2		step 3		step 4	
	Beta	P-value	Beta	P-value	Beta	P-value	Beta	P-value
*background variables*								
Gender	0.19	< 0.01	0.15	< 0.01	0.14	< 0.01	0.12	< 0.01
Residence	0.06	0.08	0.02	0.56	0.00	0.89	0.03	0.25
SES mother	0.07	0.04	0.07	0.06	0.07	0.04	0.05	0.13
SES father	0.11	< 0.01	0.08	0.02	0.06	0.06	0.07	0.02
*perceived physical-environmental*								
Availability at home			0.37	< 0.01	0.26	< 0.01	0.11	< 0.01
Availability at school			0.02	0.51	0.01	0.87	-0.01	0.75
*perceived socio-environmental*								
Modelling					0.13	< 0.01	0.08	0.02
Active encouragement					-0.05	0.30	-0.12	< 0.01
Demand family rule					0.22	< 0.01	0.22	< 0.01
Allow family rule					-0.01	0.87	-0.03	0.44
*personal*								
Knowledge							0.10	< 0.01
Attitudes							0.00	0.92
Liking							0.19	< 0.01
Self-efficacy							0.10	< 0.01
Preferences							0.22	< 0.01
Perceived barriers							-0.02	0.46
								
adjusted R^2^	0.062		0.192		0.240		0.394	
R^2 ^change	0.067	< 0.01	0.131	< 0.01	0.051	< 0.01	0.157	< 0.01

## Discussion

The results from the Icelandic part of the Pro Children survey shows that a large proportion of 11-year-old children's fruit and vegetable intake can be explained by environmental and personal factors. Environmental factors appear to be more important for children's vegetable intake than for their fruit intake. The personal factors found to be associated with fruit and vegetable intake were self-efficacy, preferences, liking and knowledge, suggesting the importance of addressing these variables in interventions.

All independent variables were significantly correlated to children's fruit and vegetable intake, with the exception of availability at school. In the present study, however, 31% of the children reported having eaten fruit in school the previous day, according to the 24-hour recall. The actual availability of fruit and vegetables at schools is low in Iceland, but as many children bring fruit with them to school, the availability at home appears to be a more important determinant of fruit and vegetable intake in Iceland. However, this could be changed by increasing the availability of fruits and vegetables at schools. In a Norwegian study it was shown that free subscription of fruits and vegetables to all pupils at school, at no cost to their parents, is an effective strategy to increase overall fruit and vegetable consumption [18]. Paid subscription to a fruit and vegetable program, however, had a limited effect on the overall fruit and vegetable intake in the Norwegian study, but a Danish subscription study showed an increase of the same magnitude of fruit intake among both non-subscribers and subscribers [19]. In the Danish study the fruit was given at school during a "fruit break", which seems to offer a positive setting for eating fruit and vegetables.

In the regression analysis, respectively 31% and 39% of the variance in children's fruit and vegetable intake was explained by the model. The predictiveness of the present model is considered good compared to similar studies. In a Norwegian study among school children (6^th ^and 7^th ^graders), 34% of the variance of the children's reported fruit and vegetable intake was explained by the measured factors [20]. In an American study among adolescents, where a comprehensive model, including numerous socio-environmental, personal and behavioural factors were tested, only 13% of the variance in fruit and vegetable intake was explained [21]. A review of the literature on models with psychosocial variables predicting dietary fat and fruit and vegetable consumption generally revealed low predictiveness of the models as they explained less than 30% of the variance, and among children and adolescents they were even less predictive [22].

The background variables of gender, residence and socioeconomic status (SES) were included in the model as these variables can be confounders [23]. Gender and the father's socioeconomic status contributed significantly to the explanation of variance of vegetable intake, while parents' socioeconomic status did not affect children's fruit intake. Gender became non-significant for fruit intake when personal variables were added to the model, which could be explained by the clear gender differences in personal factors. Social and personal variables also explain more of the variance among girls than boys; however, among boys background and environmental variables seem to explain more of the variance. Studies from other countries have shown that children and adolescents from lower socioeconomic background consume less fresh fruit and vegetables [24-27]. Socioeconomic differences have been small in Iceland, but in the last decade, in an upswing of the Icelandic economy, inequality has grown [28]. The most effective and efficient way to reach a large segment of the population is through elementary schools [29]. While the efforts to increase fruit and vegetable intake need to reach all children, they need in particular to be suitable to those at highest risk for inadequate intake. Gender must therefore to be taken into consideration, and the association between SES and fruit and vegetable consumption should be followed over time to prevent any unfortunate growing inequality in intake among low SES groups.

Availability at home was found to be one of the most important determinants of children's vegetable intake; it seems, however, less important for children's fruit intake, although still important. This is consistent with studies from other countries that have found that those having high availability/accessibility to fruit and vegetables eat more than those with lower availability/accessibility [20,21,30]. It must be noted that the environmental factors in the present study are perceived environmental factors as the data was self-reported by the children. Thus perceived availability might be increase by making fruit and vegetable more visible, for example keeping fruit in a bowl on the table instead of in the refrigerator. The availability/accessibility of fruits and vegetables has been proposed as one of the most important determinants for children's and adolescent's intake of fruits and vegetables. Higher intake of fruits and vegetables could thus be reached, by making fruit and vegetable as easily available as possible.

Social factors seem to have a similarly strong effect on both fruit and vegetable intake. Modelling or subjective norm, i.e., the perceived fruit and vegetable intake of parents and friends, seems to have a stronger effect on girls than boys. Modelling may work through personal factors for instance increasing self-efficacy, which might be the reason for modelling becoming non-significant for fruit intake when the personal factors were added to the model.

Personal factors seem to have stronger effects on fruit intake than on vegetable intake. Self-efficacy was the strongest determinant of fruit intake, and somewhat weaker effect for vegetables. Children seem confident that they can eat a sufficient amount of fruit, if they want to. They feel a bit less confident, when it comes to eating sufficient vegetables. Vegetables are more often eaten as part of a meal than between meals. It is thus more in the hands of the parents than the kids themselves and may demand more cooking skills or preparation time, although Icelandic children at this age seem to prefer raw rather than cooked vegetables [8]. It could be suggested that raw vegetable is preferred by children in the Nordic countries because they are more familiar to it. The consumption has traditionally been highest in the autumn when the vegetable is harvested, often eaten raw. In the Nordic countries there is not the same variety of vegetables as in the warmer southern countries, and in Iceland (at least) there is not the same tradition of cooked vegetable recipes (as the soup in Spain and Portugal for example). Knowledge of recommendations was also found positively related to intake. Increasing nutritional education, including skills necessary for preparing fruit and vegetable for consumption, might increase the frequency of fruit and vegetable intake.

Preferences and liking were the strongest personal predictors of vegetable intake. These factors were also predictors of fruit intake although not as strong. Preferences and liking are similar factors; however, they are measured differently. Liking was assessed by asking if they liked fruit and vegetables in general, while preferences were assessed by asking children how much they liked 12 frequently consumed fruits and vegetables. Preferences have been strongly correlated with fruit and vegetable intake in studies from other countries [20,21,30,31]. Preferences were also found to be moderators of the relationship between availability and intake [30]. Availability and accessibility, on the other hand, may be necessary for acceptance of fruits and vegetables [11,21,32]. Study using mere exposure of vegetable has shown increased liking in young children suggesting that repeatedly inviting child to taste small amount, without emphasis on how much they eat, is a good strategy to promoting liking [32], but it must be important that the quality of the vegetable is good. Reward on the other hand might be necessary to encourage children that simply refuse to taste the food at all, since the food must be tasted for the exposure to be effective [32]. The interaction between availability and preferences supports the implementation of multi-component interventions with a strong environmental component; this might be especially important in low consumption groups.

The strength of this study is the large and representative sample, with a high participation rate. The data are self-reported; therefore, all measures are perceived measures, but thorough validity and reliability studies have shown measures to be valid and reliable [13,15]. As this was a cross-sectional study, it cannot express causality between the determinants and frequency of fruit and vegetable intake. Further limitations of the study were that there were few items per scale, but in questionnaire development there is always a trade-off between precision and extensiveness [13].

## Conclusion

Interventions to increase fruit and vegetable intake among children should aim at both environmental factors such as greater availability of fruit and vegetables and personal factors as preferences, liking, self-efficacy and knowledge levels concerning nutrition. These factors interweave, and by increasing exposure, preferences and liking might be increased and thereby the intake, especially of vegetables.

## Competing interests

The author(s) declare that they have no competing interests.

## Authors' contributions

AGK worked on the statistical analysis and wrote the first draft of the manuscript and made the greatest contribution to this paper. KIK, PD and IDB participated in designing the study and project planning. IDB and MW participated in the data analysis. ITh was the local project leader and participated in all parts of the work. All authors provided critical revision of the paper, and read and approved the final manuscript.
